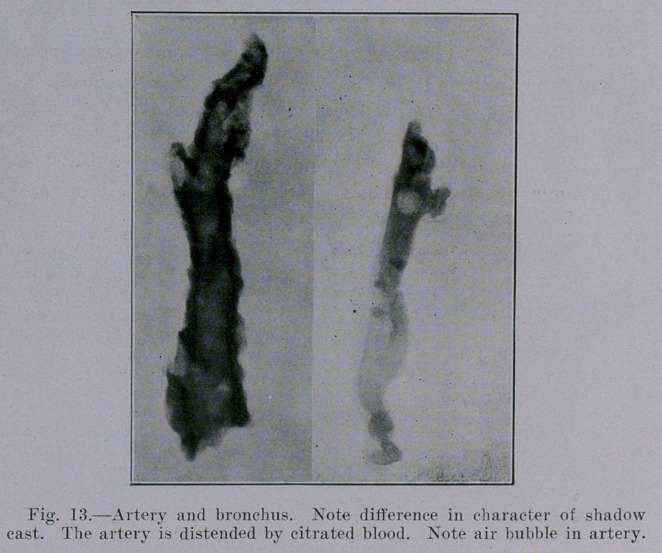# Skiagraphic Study of Thorax, Thoracic Wall and Thoracic Viscera*From the Pathological Laboratory, Austin Presbyterian Sanitarium.

**Published:** 1915-09

**Authors:** L. B. Bibb, C. E. Gilliland

**Affiliations:** Austin, Texas; Austin, Texas


### TEXAS MEDICAL JOURNAL

**Dr. F. E. Daniel, Founder. Established July, 1885**

MRS. F. E. DANIEL, - Publisher and Managing Editor

Published Monthly.—Subscription, $1.00 a Year.

VOL. XXXI AUSTIN, SEPTEMBER, 1915 No. 4

**The publisher is not responsible for views of contributors**

### Original Articles.

If there is any one thing the editor enjoys above another it is an opportunity to point out the achievements of our own Texas physicians. We are reproducing from the archives of *Internal Medicine* the following article by Drs. Bibb and Gilliland, of Austin, because we are very proud of the research work that it shows. The article has received very favorable comment by some prominent physicians in the North and East, and lest some of the Texas doctors should fail to see it we are reproducing it in our JOURNAL. Thanks are due the A. M. A. for the use of the cuts.—ED.

### Skiagraphic Study of Thorax, Thoracic Wall and Thoracic Viscera*

BY L. B. BIBB, M. D., AND C. E. GILLILAND, M. D., AUSTIN, TEXAS.

The Roentgen-ray picture of the normal or abnormal thorax invariably shows certain opacities corresponding to the hilus of the lung. Latent tuberculosis is found in this same location, and it has been stated that tuberculosis always begins here and spreads through the lung in a peripheral direction. It is of prime importance, therefore, to ascertain if possible the cause of these opacities.

An attempt was made to analyze the roentgenogram of the thorax by taking a cadaver and making a Roentgen-ray plate of the thorax intact: of the thorax with heart and lungs removed; of the posterior thoracic wall alone; of the detached heart and

*From the Pathological Laboratory, Austin Presbyterian Sanitarium.

lungs; of the heart separate; of the lungs freed from the heart and vessels, and finally of the lungs with the bronchi, artery, or vein injected with opaque emulsion of barium sulphate, or with citrated blood.

The cadaver was that of a negress, aged about 65, who died of gangrene of the legs due to extreme anasarca, probably of renal origin. No physical or other examination of the lung was made prior to death.

Fig. 1.—Intact thorax; picture made post mortem. Note the mottling seen in some of the intercostal spaces, especially on right side, low down. Heart is large, apex projecting beyond edge of plate.

The roentgenogram of the intact thorax showed an enormous heart shadow. This roentgenogram was made with the subject lying on the back, the plate underneath and the tube 26 inches above the plate.

The roentgenogram of the intact thorax does not show quite so many opacities us usual beside the heart, because the heart shadow overlaps and obscures them. Nevertheless, a few opacities appear in the region of the hilus of the lung. A careful dissection later showed complete absence of calcified lymph-nodes; the numerous peribronchial lymph-nodes were pea-sized and of a dark, almost black, color. In Figure 2 the costosternal cartilages are seen, just to the right of the sternum. It is impossible to trace these cartilages throughout their extent, and hence they may at times cause shadows that might be mistaken for shadows of something else, as, for instance, calcified nodes.

Fig. 2.—Front and bock thoracic wall with lungs and heart removed. Note absence of densities or obscurities in interspaces. Intercostal cartilages show very faintly near arrows.

The lower lobe of the right lung shows an infiltrated condition, which was evidenced in the gross specimen by a sharply demarcated zone of deep red in contrast with the pale color of the normal lung above. This infiltration shows beautifully in Figure 6. It was easily detected by palpation of the specimen, as the lung was less crepitant in the affected region. The roentgenogram of the intact thorax gives only a hint of this condition. The failure to show this might be attributed to the fact that the tube was not centered directly over the region, and might suggest that an intensive study of the lung could be better made by a series of small roentgenograms made with the tube in each instance accurately centered over the area in question.

Fig. 3.—Posterior thoracic wall alone. Intercostal cartilages absent.

The picture of this thorax does not show the bronchovascular tree, probably because the heart and pericardium overshadows the portion where the tree should show plainest, and because of the post-mortem collapse of the vessels.

A comparison of Figure 2 with Figure 1 shows that practically all the opacities are due to the contents of the thorax and not to the thoracic wall. The single exception is the shadow due to the costosternal cartilages referred to above, and which is visible in Figure 2. The blood-vessels and lymph-nodes of the chest wall do not show at all. Figure 3 is the equivalent of Figure 2, except that the anterior thoracic wall has been removed.

Figure 4 is instructive in that it shows many dense areas in the region of the hilus. These shadows are not due to lymph-

Fig. 4.—Heart and lungs after removal. Note consolidation of lower lobe of right lung. Hilus shadow shows on left side. Note the arborization of lung by bronchi and vessels.

nodes, because dissection proved that none of the nodes were calcified. It is difficult to account for all these densities on the ground that they are shadows of bronchi, because the bronchi (as will be noted later in this article) show as pairs of parallel lines with a rarefied area between. Certain it is that the bronchi do not satisfactorily account for all the hilus area. A part of the hilus density is evidently due to blood-vessels, pericardium, mediastinal fat, and adventitious connective tissue.

Figure 5 presents all the densities and opacities due to the fibrous connective tissue in and around the arteries, veins and heart. Figure 5 stands in marked contrast to Figure 6, because the latter showing the lungs alone is comparatively free from

Fig. 5.—Heart and part of great vessels. Note that the heart, connective tissue, and vessels all obstruct the Roentgen rays.

densities and opacities. It seems that the contrast between the roentgenogram of the lungs alone (Figure 6) and that of the heart and vessels (Figure 5) brings out a valuable lesson, namely, that hilus shadows are due not only to the bronchi, but also to the blood-vessels, and adventitious connective tissue.

Our studies were continued with lungs from healthy sheep. These organs are very similar to human lungs but differ in one

Fig. 6.—Lungs freed from heart and vessels. The probes are in arteries and veins, but these are collapsed and do not show. Note above all that each bronchus shows as two parallel dark lines with a light band between. Note the consolidated lower right lobe.

Fig. 7.—Heart and lungs of sheep.

rather important particular; namely, they have thin-walled bronchi with relatively large lumen. The density of bronchial shadows as compared with that of blood-vessel shadows is brought out in Figures 7 and 8. The first of these shows sheep lungs and heart intact. The second (Figure 8) shows sheep lungs with the heart

Fig. 11.—Sheep heart and lungs with vessels empty and collapsed; needles inserted ready to inject citrated blood.

removed while the blood-vessels and other little tags of mediastinal tissue remain. Note how much the picture is cleared up when the lungs are freed from the vessels (as far as possible). Figure 9 represents the lungs of the sheep after all blood-vessels and tags of mediastinal tissue are removed from the hilus. The minute density near the lower border of the left lung (seen better in Figures 8 and 10) is a calcified area, verified by actual dissection. Its occurrence in this sheep, selected at random, indicates that calcified areas are not uncommon, especially since we have found them in about 5 per cent of human chests examined.

Fig. 12.—Same as Figure 11, except clamps were released and citrated blood was allowed to run in without moving specimen, which is in almost exactly the same position as shown in Figure 11. Note the pulmonary artery shadow accompanying the primary bronchi. The arrow points to a branch of the pulmonary artery going to small upper lobe.

We do not consider a dense area as necessarily a calcified area unless it is clearly defined and isolated.

The appearance of the heart and lungs in Figures 4 and 7 is in one respect unnatural, namely, in so far as heart and vessels are practically empty. It can be observed in these two pictures that the blood-vessels do not cast a distinct shadow. The blood content is lacking. Figure 11 and Figure 12 show the difference between the shadow cast by the pulmonary artery when empty and when distended by blood. The artery with its column of blood casts a distinct shadow in Figure 12. The relative position of all structures is identical in the two pictures, because the needle for injecting the blood was *in situ* before Figure 11 was made. Immediately after exposing the plate for Figure 11 the clamps were released and citrated blood was allowed to run into the pulmonary artery under six feet gravity pressure. Note that the artery with its contained blood casts a dense shadow, whereas the accompanying bronchi show negatively as vacuities except that the side walls of the bronchi seen in section show as a density.

Fig. 13.—Artery and bronchus. Note difference in character of shadow cast. The artery is distended by citrated blood. Note air bubble in artery.

Figure 13 shows the difference in the roentgenogram of a bronchus and that of an artery of about the same size. The artery is full of blood. It will be observed that the bronchus shows as a slight streak (lumen) bordered by a dark streak (wall) on each side. The blood-vessel, however, shows as a uniformly dark band, except for the fact that in this instance an air bubble shows as a round light area.

None of the plates have been retouched, and the half-tones do not give an absolutely perfect idea of all the points.

### CONCLUSIONS.

1. The anterior and posterior chest walls offer no source of error in interpreting hilus shadows, except that the costal cartilages may be irregularly calcified; this condition is easily recognizable in the roentgenogram.

2. When the lung and heart are removed from the thorax and roentgenograms made, it is clearly seen that all the obscure densities in the thoracic roentgenogram are of visceral origin.

3. The vessels and adventitious connective tissue cast distinct shadows on the roentgenogram.

4. The shadows cast by the bronchi are linear and do not easily fuse to form a broad shadow such as that seen ordinarily in the hilus region. On the other hand, the shadows of blood-vessels are uniform, and when filled with blood might easily fuse to form the greater part of the hilus shadow.

5. The lumen of the bronchus more than compensates for the fibrous tissue in its wall, so that the bronchial shadow consists (on the negative) of a dark band bounded by two narrow light bands, the latter being due to the bronchial wall in section.

6. The vessels correspond to the bronchi and are distributed in a similar manner throughout the lung, except the first one or two subdivisions at the hilus.

7. The hilus shadow is not due to lymph-nodes, although in some cases of disease these might participate in its formation.

NOTE.—After writing the foregoing we discovered a reference to similar work done earlier by Dunham, Boardman and Wolman, who conclude that that the hilus shadow is due jointly to bronchi and vessels; also work by Fraenkel and Lorez, who attribute the entire hilus shadow to the vessels, especially the pulmonary artery; and work by Sewell and Childs, who, like us, believe that the hilus shadow is almost entirely due to the vessels. No reference was made to any injections of citrated blood. For this reason, and also for the reason that the workers mentioned did not perfectly agree among themselves, the present report is published.

### REFERENCES.

1. Riviere: Early Diagnosis of Tubercle, Oxford University Press, London, 1914.

2. Dunham, Boardman and Wolman (Phipps Dispensary): The Stereoscopic X-ray Examination of the Chest with Especial Reference to the Diagnosis of Pulmonary Tuberculosis, Bull. Johns Hopkins Hospital, 1911, xxii, 229.

3. Fraenkel, E., and Lorez, A.: The Anatomical Meaning of the Hilus Shadow in the Skiagram of the Chest, Arch. Roentgen Ray, 1910, xiv, 288.

4. Sewell, H., and Childs, S. B.: A Comparison of Physical Signs and X-ray Pictures of the Chest in Early Stages of Tuberculosis, *The Archives of Internal Medicine*, 1912, x, 45.

## Figures and Tables

**Fig. 1. f1:**
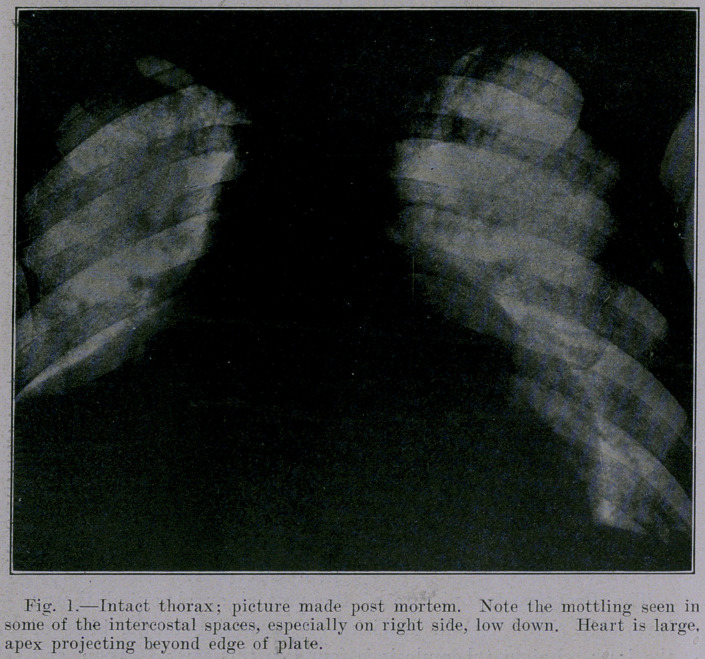


**Fig. 2. f2:**
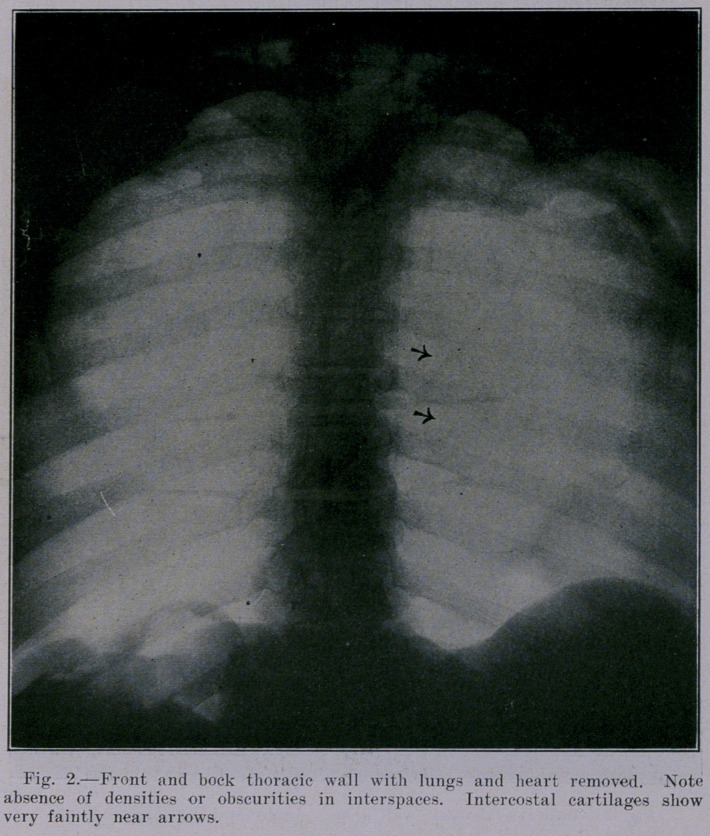


**Fig. 3. f3:**
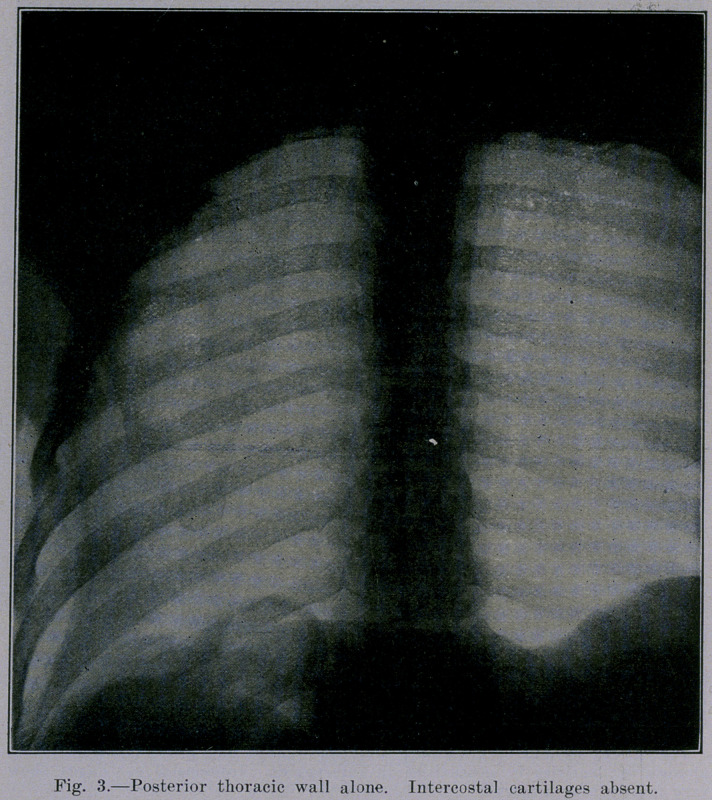


**Fig. 4. f4:**
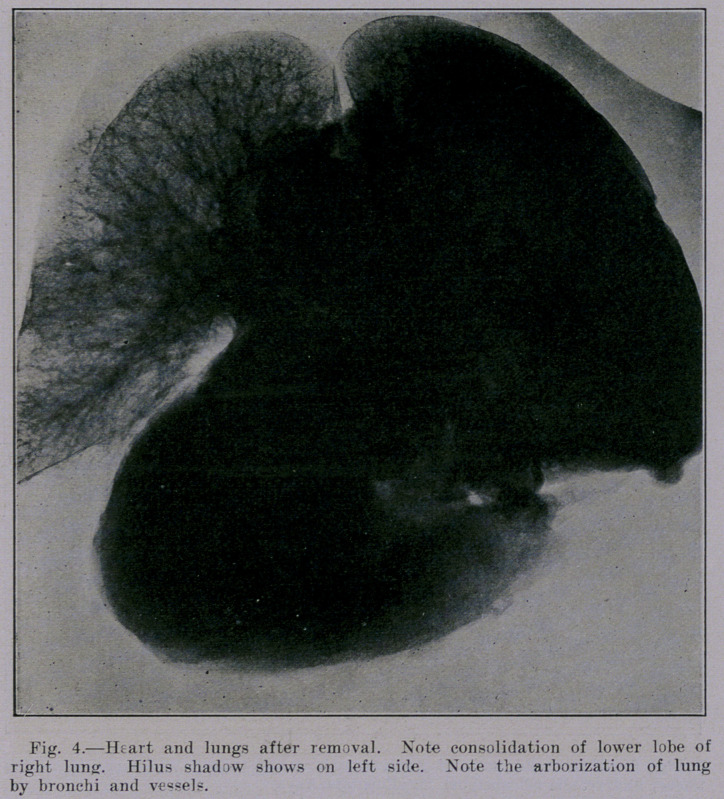


**Fig. 5. f5:**
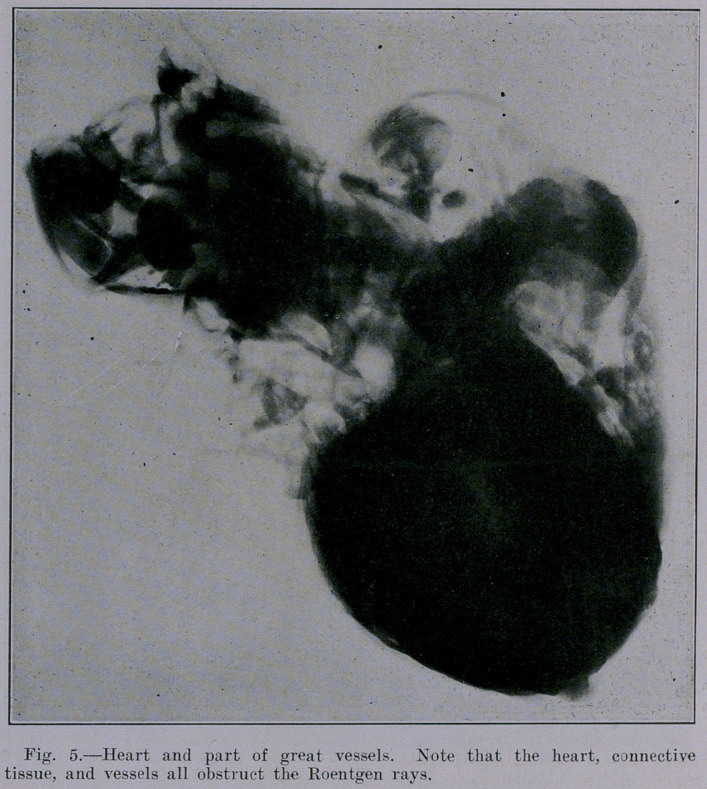


**Fig. 6. f6:**
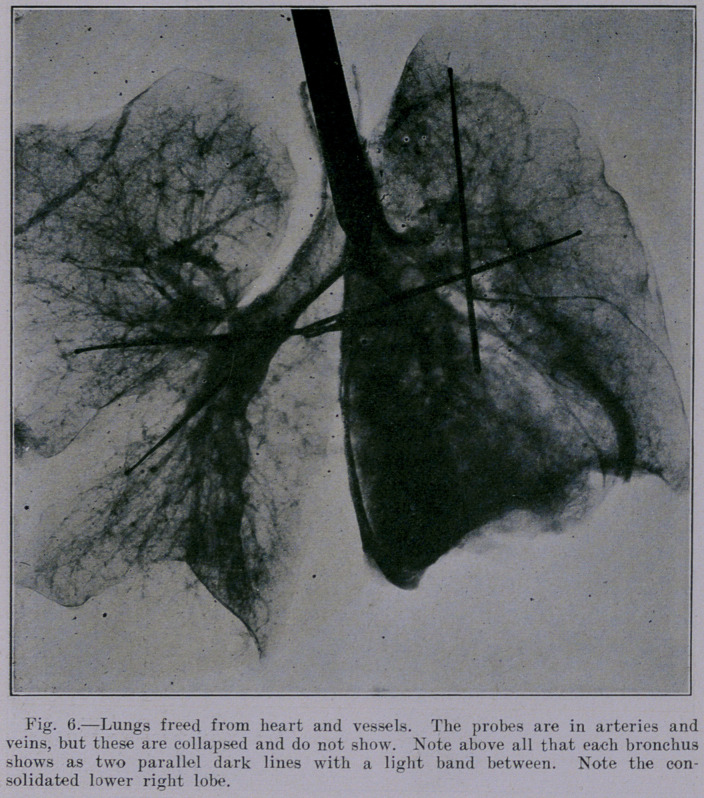


**Fig. 7. f7:**
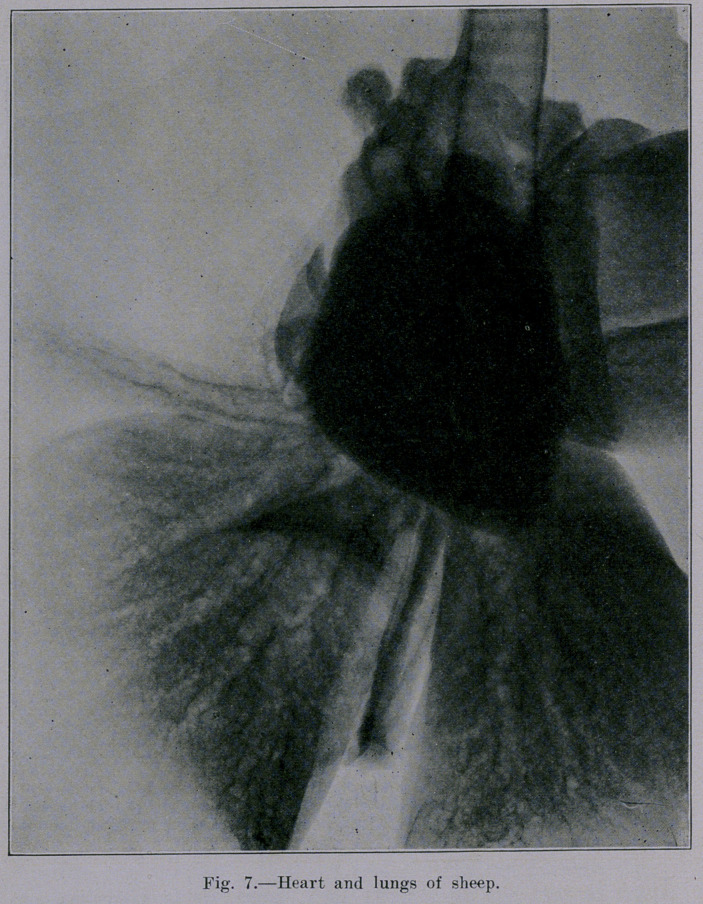


**Fig. 8. f8:**
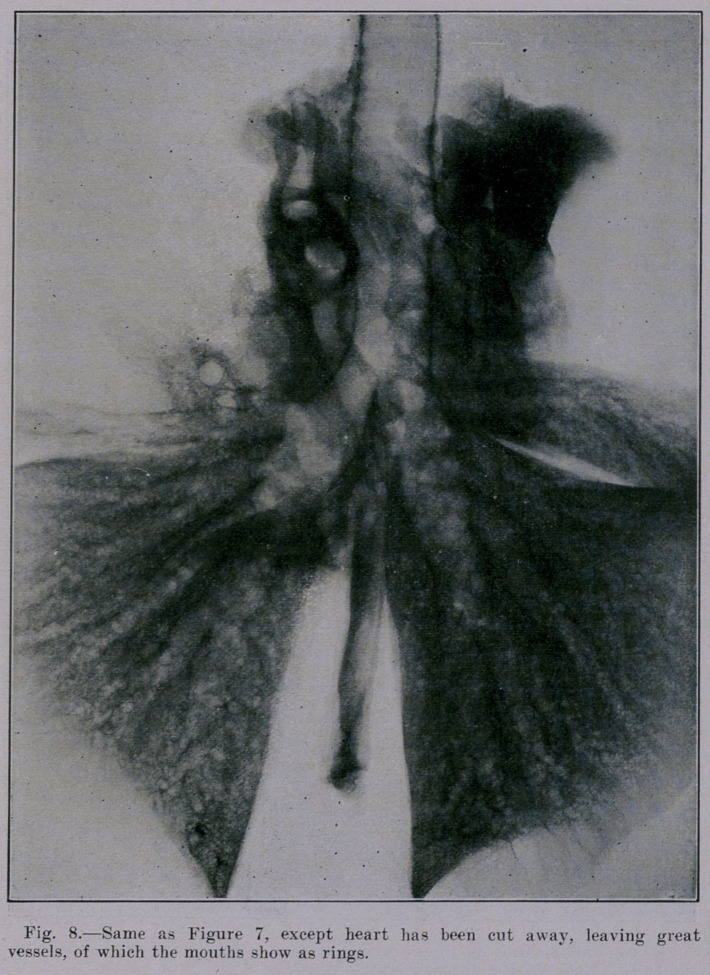


**Fig. 9. f9:**
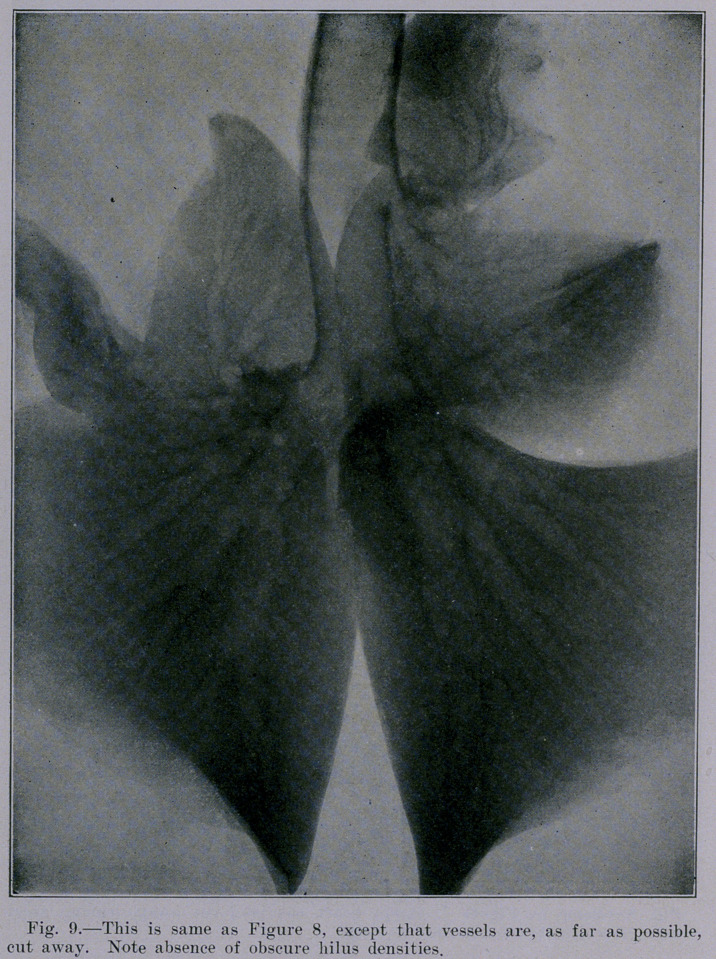


**Fig. 10. f10:**
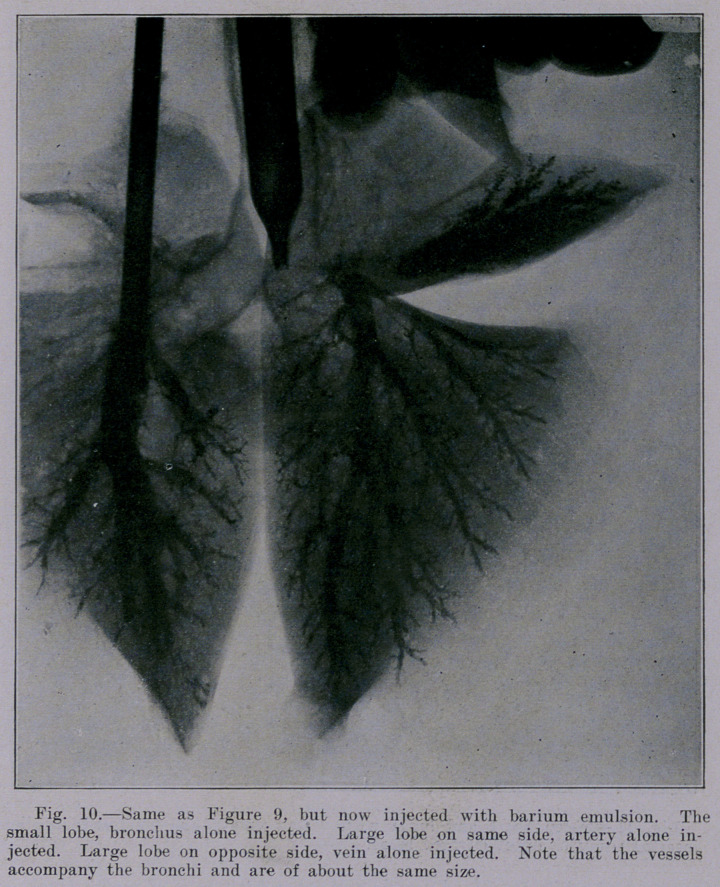


**Fig. 11. f11:**
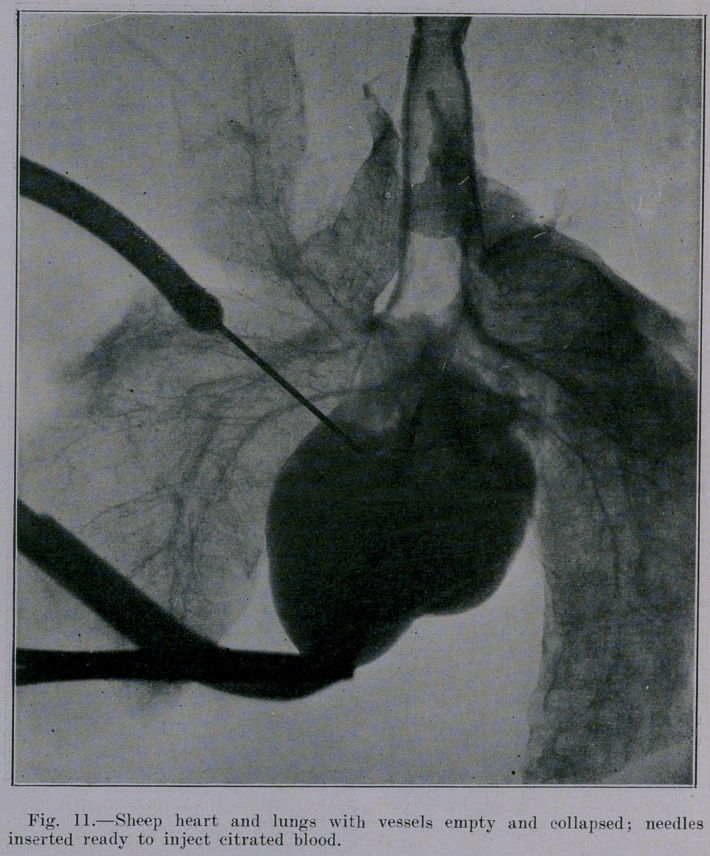


**Fig. 12. f12:**
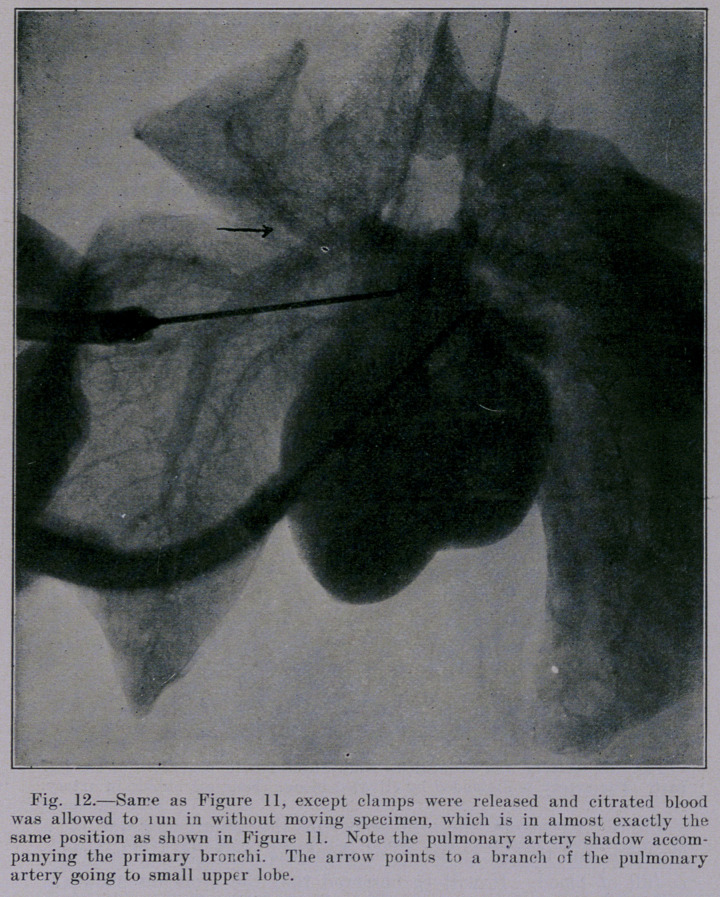


**Fig. 13. f13:**